# Delineation of the electrocardiogram with a mixed-quality-annotations dataset using convolutional neural networks

**DOI:** 10.1038/s41598-020-79512-7

**Published:** 2021-01-13

**Authors:** Guillermo Jimenez-Perez, Alejandro Alcaine, Oscar Camara

**Affiliations:** 1 PhySense research group, BCN-MedTech, Department of Information and Communication Technologies, Barcelona, 08018 Spain; 2 Facultad de Ciencias de la Salud, Universidad San Jorge, Zaragoza, 05830 Spain; 3 Biomedical Signal Interpretation and Computational Simulation (BSICoS) group, Aragón Institute of Engineering Research, Zaragoza, 50018 Spain; 4 Biomedical Research Networking Center in Bioengineering, Biomaterials and Nanomedicine (CIBER-BBN), Madrid, 28029 Spain

**Keywords:** Computer science, Biomedical engineering

## Abstract

Detection and delineation are key steps for retrieving and structuring information of the electrocardiogram (ECG), being thus crucial for numerous tasks in clinical practice. Digital signal processing (DSP) algorithms are often considered state-of-the-art for this purpose but require laborious rule readaptation for adapting to unseen morphologies. This work explores the adaptation of the the U-Net, a deep learning (DL) network employed for image segmentation, to electrocardiographic data. The model was trained using PhysioNet’s QT database, a small dataset of 105 2-lead ambulatory recordings, while being independently tested for many architectural variations, comprising changes in the model’s capacity (depth, width) and inference strategy (single- and multi-lead) in a fivefold cross-validation manner. This work features several regularization techniques to alleviate data scarcity, such as semi-supervised pre-training with low-quality data labels, performing ECG-based data augmentation and applying in-built model regularizers. The best performing configuration reached precisions of 90.12%, 99.14% and 98.25% and recalls of 98.73%, 99.94% and 99.88% for the P, QRS and T waves, respectively, on par with DSP-based approaches. Despite being a data-hungry technique trained on a small dataset, a U-Net based approach demonstrates to be a viable alternative for this task.

## Introduction

Surface electrocardiogram (ECG) is the main cardiac diagnostic and monitoring tool in clinical practice due to its widespread accessibility and ease of use. Usually, physicians perform visual inspection of the ECG in order to diagnose a patient, interpreting potential pathological deviations in the waveform. However, these markers might go unnoticed to non-specialists or even to trained cardiologists, especially when analysing multiple leads for several heart cycles or in stress-related situations. Moreover, this analysis is often reliant on the definition of a set of structured measurements in the shape of various intervals and segments of the ECG, among others^1^.

Computational methods can help unburden physicians of these problems by providing objective measurements over clinical data^2^ or by aiding in the discovery of potential biomarkers^3,4^. For these purposes, ECG detection and delineation (hereinafter delineation) is often a prerequisite step, aiding in data structuring^3^. ECG delineation consists in computing the onset and offset locations for each ECG wave (P, QRS and T waves). Delineation can be performed directly on all available leads (multi-lead) or on individual leads (single-lead).

Several computational methods exist in the literature for ECG data processing. To the best of our knowledge, digital signal processing (DSP) algorithms using the wavelet transform and rule-based adaptive thresholds are often cited as state-of-the-art for ECG delineation^5,6^, reaching high precision and recall values of 95%, 99% and 98% for the P, QRS and T waves. However, these methods require laborious rule adaptation when extended to morphologies outside the development dataset; moreover, these algorithms were fine-tuned using the whole dataset, compromising their generalization.

Although DSP algorithms have historically been used for this purpose, machine learning (ML) tools are gaining momentum for biomedical applications. Nonetheless, and in spite of their good performance, ML methods on the ECG are scarce and have mainly focused on classification^7^. As suggested by Pinto et al.^8^, this can be caused by the lack of large, manually annotated databases for ECG analysis, usually including less than a hundred patients.

Classical ML algorithms, in the shape of Gaussian mixture models^9^ or hidden Markov models (HMM)^10^, have been applied for ECG delineation. However, these methods might scale poorly when trained on large datasets and generally underperform when compared to DSP- and other ML-based algorithms. Deep learning (DL) algorithms, a branch of machine learning capable of assimilating large amounts of data, have also been used for delineation. Specifically, convolutional neural networks (CNN)^11,12^, long short-term memory (LSTM) networks^13^ and fully-convolutional networks (FCN)^14,15^. However, some of these works solely delineate the QRS wave^11^, whereas others only validate their performance on sinus rhythm^14^ or show reduced performance compared to DSP-based approaches^12,13,15^.

In this work we present the adaptation of the U-Net architecture^16^, the most successful FCN for biomedical image segmentation, for ECG delineation. For this purpose, the U-Net was adapted to one-dimensional data and delineation was framed as a segmentation task. The developed methodology was tested on the PhysioNet’s QT database^17^, which holds approximately 3,000 two-lead beats annotated by expert cardiologists having both leads in sight. Given the difficulties posed by the small dataset, with high intra-recording beat redundancy and large patient variability, several regularization strategies were applied, consisting in developing ECG-tailored data augmentation (DA) such as baseline wander or powerline noise, in performing semi-supervised pre-training with low-quality labels and in adding in-built regularizers such as Spatial Dropout (SDo) and Batch Normalization (BN) in the architecture. A large array of architectural variations were tested for completeness.

The rest of the paper is organized as follows. “Materials” section describes the employed database. “Methods” section details the methodology followed in this work. “Results” section  addresses the results obtained by this work. “Discussion” section  discusses about the obtained results and their implications on the feasibility of applying this pipeline in the clinical practice. Finally, “Conclusions” section summarizes this work’s conclusions. A preliminary version of this work has been reported in^15^.

## Materials

The QT database was employed for model training and evaluation^17^. The QT database is comprised of 105 ambulatory, two-lead recordings of 15 minutes at 250 Hz representing a variety of pathologies, comprising arrhythmia, ischemic and non-ischemic ST episodes, slow ST level drift, transient ST depression and sudden cardiac death. Two label sets exist per recording: a high-quality annotation performed by an expert cardiologist (“high-quality”, hereinafter) consisting of approximately 30 fully delineated beats per recording, and an automatic delineation (“low-quality”) performed on every beat of each recording^18^. The low-quality ground truth is produced in a single-lead manner, whereas the high-quality dataset is annotated in a multi-lead fashion. Each annotation set holds nine fiducials per beat: the P (if present), QRS and T wave detection markers and their respective onsets and offsets.

Some recordings in the high-quality dataset had to be partially re-annotated, as they contained extrasystolic beats that were neither detected nor delineated. Specifically, 112 beats in recordings *sel102*, *sel213*, *sel221*, *sel308*, *sel44* and *sel820* were added. Isolated delineations in the high-quality dataset were also excluded, as they were unusable for training the algorithm. Records *sel232*, *sel233* and *sel36* were discarded given that the annotations were incomplete. A single recording, *sel35*, was discarded due to being the only recording in atrial flutter, making it impossible to abstract this morphology with a single example. A total of 3, 246 high-quality beats and 135, 170 low-quality beats were available for training.

**Figure 1:**
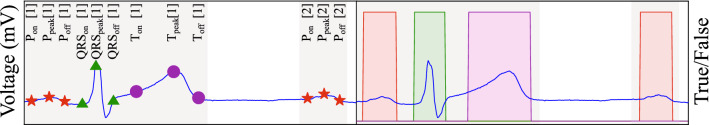
Database’s high-quality ground truth as fiducials (left) and as three overlapped binary masks (right). Red mask/stars: P wave. Green mask/triangles: QRS wave. Magenta mask/circles: T wave. The figure depicts the employed notation.

Lastly, all fiducials for a recording *r* and lead $\ell$ were transformed into binary masks *B*:

1 $$\begin{aligned} B^{(r,\ell )}[n] = \left\{ \begin{array}{ll} 1 &{} \text {if} \; n \in \left[ w^{(r,\ell )}_{\text {on}}[m], w^{(r,\ell )}_{\text {off}}[m]\right] _{0\ldots M} \\ 0 &{} \text {otherwise} \\ \end{array} \right. , \end{aligned}$$

where $W_i^{(r,\ell )}$ represent the fiducials, with $W \in \{P,\, QRS,\, T\}$, $m \in [0,M]$ are the number of annotated fiducials, and *n* is the sample number. Given the existence of three main waves, the information of the different waves was encoded into separate channels in the label tensor. Figure 1 depicts both the original fiducials and the binary masks.

A second dataset containing outflow tract ventricular arrhythmias in the 12-lead ECG^19^ was employed to qualitatively assess the fitness of the trained models, with some results reported in the Supplementary Figs. S1–S10.

**Figure 2:**
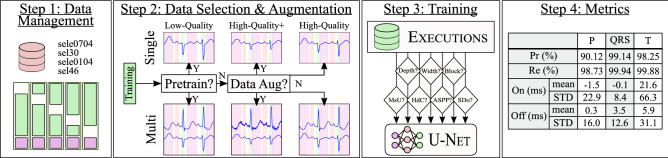
Developed pipeline. Step 1: random shuffle split of subjects (green: train, purple: test) in fivefolds. Step 2: the (single- or multi-lead) dataset is either used for training with low-quality labels or with high-quality annotations. In the latter case, ECG-tailored data augmentation is optionally applied (high-quality+). Step 3: a U-Net is instantiated per fold with a selection of execution parameters and trained with the selected data. Step 4: the fold-wise test sets are predicted and evaluated against the high-quality annotations. The employed metrics are precision and recall for detection and mean ± standard deviation (STD) of the onset and offset markers.

## Methods

The developed methodology for ECG delineation is depicted in Fig. 2. The first step describes the data splitting and management (“Data management and selection” section ). The second step outlines data selection and augmentation methodologies (“Data management and selection” and “Data augmentation” sections). The third step summarizes the base architecture and its additions (“U-Net architecture” section). The fourth step details the evaluation methodology (“Evaluation” section). The configurations tested in the third step are listed in “Experiments” section. We have made our code publicly available in https://github.com/guillermo-jimenez/ECGDelNet.

### Data management and selection

In ML procedures, data instances are usually divided into train, test and validation sets in a non-overlapping manner. However, given the high intra-recording and inter-lead beat similarity of ECG signals, a higher risk of performing an incorrect data splitting is incurred, assigning similar representations of the same entity to different sets. Models trained with this flawed splitting incur the risk of memorizing the data instead of inferring abstract patterns over it, especially in the case of high capacity models such as DL. According to Faust et al.^4^, although undesirable, this practice is widespread in ECG-based machine learning procedures. For avoiding this, fivefold cross-validation subject-wise splitting was performed.

Single-lead and multi-lead prediction strategies were attempted to address the multi-view nature of ECG. When using single-lead annotations, the algorithm would be inputted one lead at a time, producing a segmentation for every lead separately. For multi-lead prediction, a single mask would be generated when inputting all available leads as different channels. To alleviate data scarcity and low intra-recording beat variability, three different training strategies were attempted: training with high-quality data, semi-supervised pre-training with low-quality labels and applying a custom DA over high-quality labels. Semi-supervised pre-training was performed by training the model from scratch using only data annotated with an algorithm in the literature, ECGpuwave^18^, without DA. These decisions are schematized in the second step of Fig. 2.

### Data augmentation

Data augmentation improves a network’s generalization by adding realistic noise sources to the input data, learning noise-insensitive representations^20^, acting as a *de facto* regularizer. In this work, we developed six different ECG-tailored noise sources, computed to have a specific signal-to-noise ratio (SNR) with respect to an input signal, comprising additive white Gaussian noise (AWGN), random periodic spikes (RS), amplifier saturation (AS), powerline noise (PN), baseline wander (BW) and pacemaker spikes (PS):

2 $$\begin{aligned} \begin{array}{lll} \bullet \; AWGN[n] = {\mathscr {N}}\left( 0,\sqrt{{\tilde{P}}_n}\right) &{} &{} \bullet \; RS[n] = \sqrt{\frac{{\tilde{P}}_n}{f}} \sum \limits _{k = -\infty }^{k = \infty } (\delta * Sp)\left[ n - k \frac{1}{f}\right] \\ \bullet \; AS[n] = \left\{ \begin{array}{ll} - x[n] + S_v &{} if \, x[n] \ge S_v \\ - x[n] - S_v &{} if \, x[n] \le - S_v \\ 0 &{} otherwise \end{array} \right. &{} &{} \bullet \; PS[n] = \left\{ \begin{array}{ll} \sqrt{\frac{{\tilde{P}}_n}{f}} &{} if\,n \in QRSon \\ 0 &{} otherwise \end{array} \right. \\ \bullet \; PN/BW[n] = \sqrt{2 {\tilde{P}}_n} \cos {\left( \frac{2\pi f}{f_s} n\right) }, &{} \end{array} \end{aligned}$$

where ${\mathscr {N}}$ is the normal distribution, ${\tilde{P}}_n = P_s/10^{\text {SNR}/10}$ is the noise power, $P_s$ is the input signal power, $f_s$ is the sampling frequency, $S_v = p \max {\left| \mathbf{x } \right| }$ is the saturation value, $(a * b)[n]$ indicates the convolution operation, $Sp = [0,0.15,1.5,-0.25,0.15]^T + {\mathscr {U}}(-0.25, 0.25)$ is a custom filter with uniform noise that models pacemaker spikes and $\delta$ is the impulse function.

**Figure 3:**
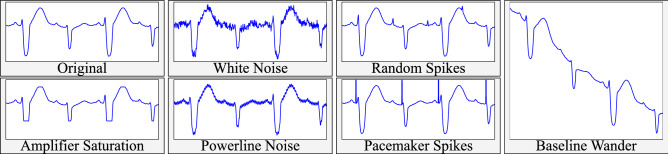
Data augmentation strategy example for an ECG recording. Re-execution results in slight signal-to-noise ratio (SNR) and frequency (*f*) variations, altering the final shape of the computed noise.

The first five noise sources were engineered to represent usual and observed variations in the dataset. Pacemaker spikes were designed to avoid misidentifying spike-like noise near QRS complexes and for completeness. Powerline and baseline noises share the same formulation but are instantiated with different hyperparameters ($f = 50Hz$ and $f = 0.5Hz$, respectively). Some noise in the generating hyperparameters was added upon generation for maximizing input variability, given $p^{(i)} = p + {\mathscr {U}}(\pm SNR/10)$, where ${\mathscr {U}}$ is the uniform distribution. Figure 3 depicts an example of the developed noise sources.

**Figure 4:**
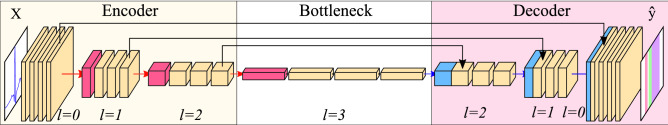
Base U-Net instantiated with 4 levels and 3 convolutional blocks per level. Blocks represent output tensors, whereas arrows indicate operations. Yellow: convolutions; red: pooling; blue: upsampling; black: concatenation. Convolutional operations extract $2^\textit{l} N$ channels per level, whereas pooling and upsampling have a kernel size of 2.

### U-Net architecture

The employed architecture is based on the U-Net^16^, which consists in an encoder, a bottleneck and a decoder with skip connections between the encoder and the decoder, as seen in Fig. 4. The encoder extracts increasingly abstract representations of the input data through several levels of stacked convolutional operations and downsampling blocks. The decoder recovers information from the bottleneck, the nexus between the encoder and the decoder, through convolutional and upsampling blocks. Skip connections allow for crisper segmentation at the object boundaries by direct information transmission from the encoder. In the U-Net, the number of convolutional filters is doubled after each downsampling block and halved after each upsampling block. For a clearer exposition, we have grouped operations in the U-Net architecture into “blocks”, which form “levels”. We define a “block” as an ordered composition of operations on a tensor $\mathbf{x }$ and a “level” as a set of operations whose results have compatible tensor size. The considered blocks are convolutional, downsampling, upsampling, and skip connection blocks. The ordering of operations in the blocks were defined to agree with the image segmentation literature^21,22^.

In this work, convolutional (*C*) and separable convolutional (*S*) operations were considered, paired with ReLU non-linearities (*NL*) and regularizers (*R*) and point-wise additions $A(\cdot , \cdot )$. The following blocks were independently explored:

$$\begin{aligned} \begin{array}{llllllllll} \bullet &{} ``Vanilla'' &{} \mathbf{y } = \;\;\;\;C(R(N(C(R(N(\mathbf{x })))))) &{} ^{16} &{} \qquad \bullet &{} Residual &{} \mathbf{y } = A(C(R(N(C(R(N(\mathbf{x })))))), \mathbf{x }) &{} ^{22}\\ \bullet &{} XCeption &{} \mathbf{y } = A(S(R(N(S(R(N(\mathbf{x })))))), \mathbf{x }) &{} ^{21} \end{array} \end{aligned}$$

To comply with the requirements posed by ECG data and the task at hand, the U-Net convolutions were replaced by 1D operations, zero padding was applied to keep input resolution, and a stem (one extra convolutional module right after the input) was included to mimic classification architectures^21,23,24^. Lastly, given an initial model testing phase, the need for stronger regularization than batch normalization was apparent. We opted to apply SDo^25^, which randomly drops entire tensor channels during training, as opposed to standard dropout, where neurons are dropped in an unstructured manner, as well as semi-supervised pre-training and data augmentation, described in “Data management and selection” and “Data augmentation” sections, respectively.

### Evaluation

The evaluation is inspired by the metrics used in state-of-the-art DSP-based algorithms^5^ for comparison purposes. A correspondence matrix *H* of the correspondence between the true (*w*) and predicted (${\hat{w}}$) wave fiducials can be computed as:

3 $$\begin{aligned} H_{ij}^{(r,\ell )} = \left\{ \begin{array}{lll} 1 &{} \text {if} &{} ({\hat{w}}_{\text {fid}}^{(r,\ell )}[j] \in [w_{\text {on}}^{(r,\ell )}[i], w_{\text {off}}^{(r,\ell )}[i]]) \\ &{} \text {or} &{} (w_{\text {fid}}^{(r,\ell )}[j] \in [{\hat{w}}_{\text {on}}^{(r,\ell )}[i], {\hat{w}}_{\text {off}}^{(r,\ell )}[i]]) \\ 0 &{} {\text {otherwise}} \end{array} \right. , \end{aligned}$$

where *r* is the subject’s recording, $\ell$ is the lead within the recording, $w_{\text {fid}}$ (with $\text {fid} \in \{\text {on, peak, off}\}$ and $w \in \{\text {P, QRS, T}\}$) are the onset, peak and offset information for a specific wave, and $i \in [0,M]$ and $j \in [0,{\hat{M}}]$ are the total true and predicted fiducials, respectively.

**Table 1:** Performance gain comparisons of design decisions, expressed as median difference values in F1 score (%), onset and offset error (ms).

	Spatial dropout			Semi-supervised pre-training			Data augmentation		
Metric	$\Delta$F1	$\Delta$Onset M±SD (ms)	$\Delta$Offset M±SD (ms)	$\Delta$F1	$\Delta$Onset M±SD (ms)	$\Delta$Offset M±SD (ms)	$\Delta$F1	$\Delta$Onset M±SD (ms)	$\Delta$Offset M±SD (ms)
P	+ **1**.**98** %	− 2.79 − 0.83	− 2.07 − 1.18	+ 1.85 %	−** 0**.**18** − **4**.**31**	− 0.64 − 2.51	+ 1.22 %	+ 0.86 − 2.10	−** 0**.**42** − **3**.**23**
QRS	+ **3**.**27** %	−** 0**.**73** − **1**.**43**	+**1**.**88** − **2**.**90**	+ 1.07 %	+ 0.08 − 0.74	− 0.23 − 1.56	+ 0.63 %	+ 0.26 − 0.90	+ 0.61 − 1.01
T	+ **7**.**21** %	+ 8.06 + 3.90	− 2.81 − 1.32	+ 1.65 %	+**1**.**53** − **3**.**88**	−** 0**.**36** − **4**.**87**	+ 0.80 %	+ 3.70 − 0.39	− 0.90 + 3.63

A positive F1 score indicates a performance increase of the design decision, whereas negative onset/offset mean or STD errors indicate more precise fiducial location. Bold values represent best independently performing approaches.

The individual lead information for a specific wave is then combined into a single correspondence matrix through a logical “OR” operator ${\bar{H}} = OR(H^{(0)}, \ldots , H^{(L)})$. The true positives (TP) for a given recording *r* were considered as $TP_r = \sum {\bar{H}}_{ij}$. False-positives (FP), on their behalf, are elements of $\tilde{\mathbf{w }}_{\text {fid}}^{(r,\ell )}$ that did not correspond to any true fiducial ($FP_r = {\hat{M}} - card(\{(i,j) \mid {\bar{H}}_{ij} = 1 \})$). Finally, a false-negative (FN) is considered when the ground truth displays a beat that is not captured by a TP (corresponding to $FN_r = M - card(\{(i,j) \mid {\bar{H}}_{ij} = 1 \})$). The precision (*Pr*) and recall (*Re*) for each *r* and $\ell$ were computed, reporting in this work the overall performance for all recordings and leads for conciseness. Additionally, the F1 score is also computed for comparing different architectural variations, as a single figure of merit.

The delineation metrics were computed for the TPs (cases where ${\bar{H}}_{ij} = 1$), as no onset/offset correspondences between the GT and the prediction exist otherwise. The relative error of the segmentation was computed through the mean and standard deviation (SD) of the difference of the actual and predicted onsets or offsets of the correspondences found in Eq. (3):

4 $$\begin{aligned} \min _{i, j, \ell } \;\; w_{\text {fid}}^{(r,\ell )}[i] - {\tilde{w}}_{\text {fid}}^{(r,\ell )}[j] \;\;\;\;\;\;\; \text {s.t.} \; {\bar{H}}_{ij} = 1. \end{aligned}$$

For comparison purposes, the delineation errors were compared to the within-dataset bias, in the shape of two different sources of error: the inter-observer and the inter-lead variability. The first accounts for the difference in criteria used by the first observer ($O_1$) and the second ($O_2$) when annotating a sub-set of the input data. The latter accounts for the difference in criteria used by one observer when delineating morphologically similar beats. For this purpose, a running cross-correlation (normalized so that autocorrelations at displacement 0 equal 1) between all pairs of delineated waves was computed for all possible overlapping positions. Those pairs that shared a 99% or higher similarity were marked as true positives (for the comparison) and accounted for the delineation error. For producing a fair comparison, a window of 40 ms in the onset and offset of the compared delineations was added.

### Experiments

For testing the model’s robustness, this work features a series of variations on the data level and in the network’s topology. Data-level variations aimed at alleviating data scarcity through the application of in-built SDo regularization ($p = 0.25$) and batch normalization (“U-Net architecture” section), DA strategies (“Data augmentation” section) and semi-supervised pre-training (“Data management and selection” section) for both single- and multi-lead inference strategies. Given our limited computational budget, no DA was applied during semi-supervised pre-training.

**Table 2:** Precision and recall ($\%$; top) and onset and offset errors (mean, M ± standard deviation, SD; bottom) of our best performing single-lead model as compared to other approaches.

		Precision (%)							Recall (%)						
		Single-lead	Multi-lead	^5^	^11^	^12^			Single-lead	Multi-lead	^5^	^11^	^12^		
						10 ms	50 ms	150 ms					10 ms	50 ms	150 ms
P		90.12	**94**.**17**	91.03	N/A	79.6	84.6	90.0	98.73	94.70	**98**.**87**	N/A	86.8	92.2	98.1
QRS		99.14	99.40	**99**.**86**	N/A	93.0	98.5	99.9	**99**.**94**	99.28	99.80	N/A	92.2	97.7	99.1
T		**98**.**25**	96.36	97.79	N/A	80.2	87.4	97.7	**99**.**88**	99.09	99.77	N/A	80.7	87.9	98.3

		Onset Error, ms							Offset Error, ms						
		Single-lead	Multi-lead	^5^	^11^	^12^			Single-lead	Multi-lead	^5^	^11^	^12^		
						10 ms	50 ms	150 ms					10 ms	50 ms	150 ms
P	Mean	**1**.**54**	− 1.72	2.0	N/A	N/A	N/A	N/A	**0**.**32**	4.01	1.9	N/A	N/A	N/A	N/A
	STD	22.89	17.83	**14**.**8**					15.99	16.08	**12**.**8**				
QRS	Mean	$-$ **0**.**07**	− 3.83	4.6	− 2.6	N/A	N/A	N/A	3.64	5.39	**0**.**8**	4.4	N/A	N/A	N/A
	STD	8.37	14.64	**7**.**7**	10.8				12.55	16.77	**8**.**7**	15.2			
T	Mean	21.57	**19**.**10**	N/A	N/A	N/A	N/A	N/A	4.55	9.93	$-$ **1**.**6**	N/A	N/A	N/A	N/A
	STD	**66**.**29**	66.51						31.11	46.33	**18**.**1**				

Sodmann et al.^12^ reports results for different window sizes (10, 50, 150 ms), considering a true positive if their prediction is contained within the window. N/R stands for “not reported”.

For each of the data-level variations, a set of topological changes were independently tested. These changes took shape in the type of convolutional block employed (“vanilla”, residual, XCeption), the network’s depth ($L \in [4, 5, 6, 7]$) and the number of convolutional blocks per level ($CB \in [ 2, 3, 4, 5, 6 ]$) were attempted. In total, 201 executions were performed testing various configurations, with training times ranging from 6 hours for the smallest models without pre-training to several days. The executions were performed in a high performance computing environment where each configuration was assigned to a single NVIDIA 1080Ti or NVIDIA Titan Xp GPU. To ensure reproducibility, the same random seed was employed in all executions. Some aspects were kept constant in all executions, such as the nonlinearity (ReLU for all blocks and sigmoid for the last block), convolutional kernel (3) and pooling (2) sizes, loss function (Jaccard), optimizer (Adam^26^) and random seed (1234).

## Results

### Model selection

This section describes the performance comparisons of independent design decisions tested in “Experiments” section. The pipeline mainly benefited from the application of SDo regularization approach, reaching improvements of 1.98%, 3.27% and 7.21% F1 score in the detection of the P, QRS and T waves, and a reductions of $-2.79 - 0.83$ ms, $-0.73 \pm 1.43$ ms and $8.06 + 3.90$ ms in onset error and of $-2.07 - 1.18$ ms, $+1.88 \pm 2.90$ ms and $-2.81 - 1.32$ ms in offset errors in the P, QRS and T waves, respectively. Such generalized improvement is also seen in semi-supervised pre-training and when using DA. Summarized results can be visualized in Table 1. The model performance degraded consistently at higher capacity models (6 and 7 levels of depth and over 4 blocks per level) whenever SDo was not applied, but performed very similarly to other model definitions when it was. Other additions such as the type of convolutional block, width and depth of the network showed comparable performance throughout all executions. A more comprehensive list of design decision performance comparisons can be seen in Supplementary Tables S1–S39.

### Best performing model

Both single-lead and multi-lead best performing models feature strong regularization techniques in the shape of pre-training with low-quality labelled data and SDo of 25%. The best performing single-lead model, in accordance to the results expressed above, consists in a model with 5 levels and 3 blocks per level employing the “vanilla” convolutional block, with P, QRS and T wave precisions of 90.12%, 99.14% and 98.25% and recalls of 98.73%, 99.94% and 99.88% for detection. The delineation performance shows errors of $1.54 \pm 22.89$ ms, $-0.07 \pm 8.37$ ms and $21.57 \pm 66.29$ ms in the onset and of $0.32 \pm 4.01$ ms, $3.64 \pm 12.55$ ms and $4.55 \pm 31.11$ ms in the offset for delineation, with Dice scores of 88.99%, 92.05%, 88.40% for the P, QRS and T waves, respectively. This model was used to predict some registries in^19^, shown in Supplementary Figs. S1–S10.

**Figure 5:**
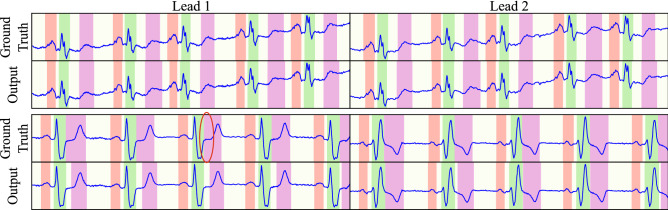
Examples of correctly predicted samples, depicting samples from sudden cardiac death (top) and ST change (bottom). Red mask: P wave. Green mask: QRS wave. Magenta mask: T wave. Representative examples have been encircled.

**Figure 6:**
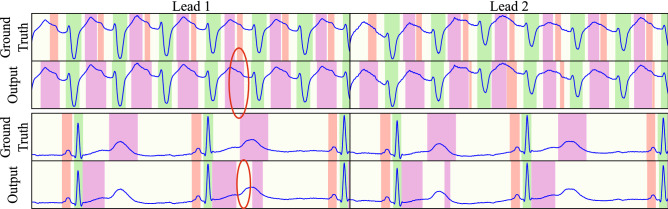
Examples of incorrectly predicted samples, featuring fused T and P waves (top) and severe bradycardia (bottom). Red mask: P wave. Green mask: QRS wave. Magenta mask: T wave. Representative examples have been encircled.

**Figure 7:**
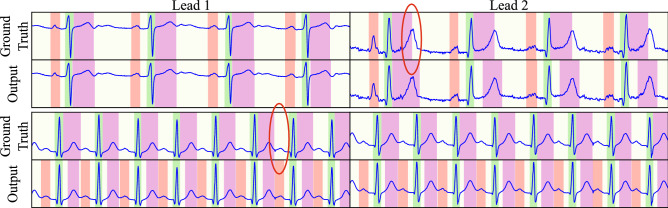
Examples of incorrectly annotated ground truth, demonstrating incorrect T offset location (top) and missed P waves (bottom). Red mask: P wave. Green mask: QRS wave. Magenta mask: T wave. Representative examples have been encircled.

The best multi-lead model features 4 levels and 6 blocks per level employing the “XCeption” convolutional block, reaching P, QRS and T precisions of 94.17%, 99.40% and 96.36% and recalls of 94.70%, 99.28% and 99.09% for detection. The delineation performance deviated from the ground truth $1.54 \pm 22.89$ ms, $1.54 \pm 22.89$ ms and $1.54 \pm 22.89$ ms in the onset and $4.01 \pm 16.08$ ms, $5.39 \pm 16.77$ ms and $9.93 \pm 46.33$ ms in the offset, reaching Dice scores of 88.19%, 92.14%, 89.33% for the P, QRS and T waves, respectively. The optimal network configuration for both single- and multi-lead is detailed in Table 2, whereas Figs. 5, 6 and 7 depict several samples from the single-lead and multi-lead approaches.

### Inter-observer and intra-observer bias

The comparison of the different sources of bias within the database have been summarized in Table 3. The inter-observer bias shows larger or comparable bias for both single- and multi-lead scenarios in the QRS onsets (3.84 ± 14.17 ms vs. -0.07 ± 8.37 ms and -3.83 ± 14.64 ms) and offsets (2.74 ± 16.94 ms vs. 3.64 ± 12.55 ms and 5.39 ± 16.77 ms). The T wave is generally delineated more precisely by the human operator in its onsets (-9.52 ± 44.85 ms vs. 21.57 ± 66.29 ms and 19.10 ± 66.51 ms) and offsets onsets (5.84 ± 39.84 ms vs. 4.55 ± 31.11 ms and 9.93 ± 46.33 ms).

The intra-observer bias, on the other hand, is consistently larger in the database as compared to the model’s predictions in the P onset (2.26 ± 42.93 ms vs. 1.54 ± 22.89 ms and -1.72 ± 17.83 ms), P offset (7.76 ± 23.28 ms vs. 0.32 ± 15.99 ms and 4.01 ± 16.08 ms), QRS onset (2.56 ± 22.95 ms vs. -0.07 ± 8.37 ms and -3.83 ± 14.64 ms), QRS offset (2.02 ± 21.9 ms vs. 3.64 ± 12.55 ms and 5.39 ± 16.77 ms) and T onset (-51.96 ± 105.88 ms vs. 21.57 ± 66.29 ms and 19.10 ± 66.5 ms1) for both single- and multi-lead scenarios, respectively. The T offset in the multi-lead prediction strategy is the only metric that shows worse performance with respect to the inherent dataset bias (4.53 ± 42.71 ms vs. 4.55 ± 31.11 ms and 9.93 ± 46.33 ms), and is within 1 sample (4 ms) difference.

**Table 3:** Onset and offset errors (mean, M ± standard deviation, SD) of the inter-observer (**a**) and intra-observer (**b**) bias within matching delineations in the ground truth.

	Onset Error		Offset Error			Onset Error		Offset Error	
	M (ms)	STD (ms)	M (ms)	STD (ms)		M (ms)	STD (ms)	M (ms)	STD (ms)
(a) Inter-observer bias					(b) Intra-observer bias				
P wave	N/A	N/A	N/A	N/A	P wave	2.26	42.93	7.76	23.28
QRS wave	3.84	14.17	2.74	16.94	QRS wave	2.56	22.95	2.02	21.9
T wave	-9.52	44.85	5.84	39.84	T wave	-51.96	105.88	4.53	42.71

No P wave annotations were produced by the second observer.

## Discussion

Deep learning techniques show improved performance upon classical approaches for supervised tasks given sufficient training data^27^. These models can be used to improve and automate tasks in the medical domain such as image (or signal) segmentation, aiding in clinical decision-making^28^. Moreover, these models can be re-trained on newly acquired data to produce a positive feedback loop that enhances performance upon usage.

Under this context, this work presents a FCN-based approach for ECG delineation by framing the problem as a segmentation task. Our work exhibits good detection and delineation performance, with metrics comparable to DSP-based methods while presenting competitive advantages and increased performance over ML-based works. A summary of the performance comparison to other approaches can be seen in Table 2. The network has an excellent detection performance, in both single-lead and multi-lead scenarios, even if trained on a small dataset. The model was thoroughly explored in its hyperparameters to assess its performance under different training conditions. Many of these model variations showed inconsistent performance gains. Only explicit regularization strategies such as SDo, pre-training on low-quality labels and DA consistently improved overall detection and delineation performance, increasing F1 scores and decreasing onset and offset errors. These variations demonstrated good detection and delineation performance in all executions.

We have compared our method with state-of-the-art methods in either DSP and ML methods. DSP-based approaches, such as Martínez et al.^5^ provide a high delineation performance and are considered state-of-the-art. Our best performing approach performed on par with these methods in detection (Table 2-top^5^) with differences in precision and recall lesser than 1%. Our model, however, produced higher delineation errors compared to the state-of-the-art, especially in the T wave (Table 2-bottom). The multi-lead approach showed consistently worse performance at delineation, with up to 15 ms difference in the T wave offset, which can be explained in two ways. Firstly, our approach is data-driven, so any bias in the QT database will be learnt by the network. Therefore, the inter-observer and intra-observer bias were computed (Table 3), demonstrating that our error was below in general (or comparable in T offset) to the intrinsic data variability. Some examples of extreme intra-observer criterion differences are shown in Fig. 7. Moreover, it is noteworthy that one mere sample difference is equivalent to 4 ms error, so large errors are equivalent to small sample differences.

Secondly, the results reported by^5^ do not allow for testing generalizability on other datasets, as they adjusted their algorithm to produce globally minimal metrics on the whole dataset without using a separate test set, thus their method’s generalizability remaining untested^1^. A fair numerical comparison between the approaches is challenging, since the proposed DL-based method produces metrics on data that the algorithm has not been previously trained on (cross-validation) and, therefore, no global error minimization is made. The used training method, however, hints at the ability of our algorithm to generalize on unseen data –which is a more desirable scenario. Although no ground truth for comparison exists, the generalizability of our approach can be seen in the prediction of some registries from^19^, a challenging database for the prediction of outflow tract ventricular arrhythmias (Supplementary Figs. S1–S10).

Overall, our approach provides comparable results to DSP-based methods, in spite of being trained on small amounts of highly biased annotations, while applying cross-validation with strict subject-wise splitting for ensuring generalization and obtaining smaller delineation errors than those in the dataset. On the other hand, DSP-based algorithms require laborious rule re-calibration when extended to other morphologies, which is heavily time consuming. In this sense, DL-based approaches such as the proposed model can more easily assimilate newly annotated data to enhance delineation performance and shorten development once the right design decisions have been modeled, thus arising as an alternative to DSP-based methods with great potential.

In contrast with DSP-based approaches, DL methods use a variety of architectures, providing a good framework for comparison. It is noteworthy, however, that the majority of the compared literature does not detail how the train/test splitting is made, leading to potentially misleading model performance, as noted in “Data management and selection” section. In general, the proposed method clearly outperforms all other data-driven approaches found in the literature, obtaining clearly higher detection values and lower delineation errors. In the following paragraphs, the different works are analyzed, grouped by type of network.

Although limited bibliography of CNN-based delineation methods exists, these compare unfavourably to FCN-based approaches. Camps et al.^11^ delineated solely the QRS wave, neglecting P and T waves, while attaining delineation performance of $-2.6 \pm 10.8$ ms and $4.4 \pm 15.2$ ms for the QRS onset and offset. The authors did not report precision or recall metrics, difficulting direct performance comparison. Sodmann et al.^12^ directly predicted the fiducial’s sample of occurrence (*w*) through fully convolutional layers. However, their work suffers from performance pitfalls, achieving differences in performance up to 10% with respect to DSP-based approaches even with large ($\sim$50 ms) tolerance windows, while disregarding detections with error higher than 250 ms. Moreover, the authors excluded 23 recordings of the QT database.

A single recurrent formulation employing LSTM has been proposed in the literature by Abrishami et al.^13^. However, their work featured relatively low precision for the QRS and T waves (94% and 90%, respectively) and overall low recall (91%, 94% and 91% for the P, QRS and T waves, respectively), and the authors did not report delineation performance metrics. This work, however, merits from having performed subject-wise splitting.

Lastly, Tison et al.^14^ published a U-Net based model for the delineation of 12-lead ECGs, similar to our initial attempt published in^15^. Tison et al. presented an asymmetric U-Net which featured an appended structure at the base level for producing 12-lead fusion and direct 8-fold upsampling from level 5 to level 2 in the decoder. The authors reported a high Dice score (P wave: 91 ± 3 %; QRS wave: 94 ± 4 %; T wave: 92 ± 5 %). The authors, however, employed a private ECG database, discarded recordings with large errors in a downstream task and heavily rely on HMM-based post-processing for refining the results. Moreover, their work is restricted to sinus rhythm recordings, compromising its generalizability on harder-to-delineate pathological beats. Although direct comparison is difficult in this case, our network needs no post-processing, has been tested against a standard database and was based on a well-founded architecture.

Besides the competitive delineation performance, we learnt several lessons for processing ECG data with DL-based techniques. Firstly, when working with ECG data, strong regularization techniques such as SDo and DA are of utmost importance, as the network easily overfitted the training data on the first epoch while stagnating the validation loss. Secondly, semi-supervised pre-training tags provides largely increased performance, plausibly due to the larger input data variability. Given the scarcity of manually annotated data in the QT database, low-quality data can give sufficient samples to learn better abstractions, acting as a regularizer via data^29^. Finally, the application of ECG-based DA methodologies seemed to increase overall performance of the network, when access to a larger database or to low-quality labelling is not possible.

An especially interesting result is drawn from the comparison of the model performance when producing single-lead and multi-lead predictions. The multi-lead fiducial computation suffers $1.89\%$ and $4.03\%$ drops in T wave precision and P wave recall, as well as large (up to 15.22 ms of difference) in onset and offset standard deviations. These gaps are partially due to the employed evaluation method, which compares the ground truth to the best predicted fiducial, irrespective of the lead on which it has been produced. This methodology, adapted from^5^ for comparison with DSP-based methods, is a double-edged sword: while it decouples the performance of the delineation to the specific lead fusion strategy, it also masks the error it would inevitably produce. A second reason for the difference in performance is that the multi-lead scenario has half the samples to learn a representation from an input space that is doubly as large, as two leads are used as input.

A last point raised by the results, the model model performance degradation on higher capacity models, can be explained by the great imbalance between the network’s capacity and the small amount of annotated data. This imbalance makes it more plausible to fall into local minima without stronger regularization techniques. In this sense, this work adds to the growing evidence that prior knowledge imposition, such as the applied regularization strategies (SDo, DA, pre-training) can be more effective than architectural modifications.

Although our network compares positively to other methods in the literature, many can be improved upon. One of the main limitations of this work is the lack of more up-to-date databases, containing a higher variability for a wider array of pathologies. Despite ECG usually being the first information registered of the patient’s cardiac condition, not many large annotated databases for ECG delineation exist. This might partially explain the systematic errors in the T wave in a reduced amount of recordings, where the network predicted rises and falls as independent waves for very long T waves (Fig. 6). With the current database, counting with 105 different represented ECG morphologies, our approach remains a proof of concept. This data scarcity could be alleviated through the application of clever data techniques, such as further semi-supervised on large, unannotated databases^30^ or through realistic simulation of ECG traces.

Besides the apparent database-related shortcomings, some improvements could be made in the architecture. Temporal dependencies explicitly modeled with RNNs^31^ or attention-based models^32,33^. Efficiency-based modifications could also be explored, such as MobileNet^34^, and model compression^35^ for deploying the model in CPU-only computers. Finally, domain-specific prior imposition, model pre-training or alternative segmentation loss development would further improve performance. Other DA schemes such as varying the heart rate, isoelectric line, specific wave shapes (e.g. voltage or width of P, QRS or T waves within a beat), DA hyperparameter tuning and composing new beats from components would also help, but the executions were made to keep an assumable computational budget.

## Conclusions

Despite its potential, DL for cardiac signal analysis is not well established in the community^1,3^. Some influencing factors are the lack of large-scale, quality databases (such as UK BioBank in the imaging community), lack of digital support (many hospitals still print ECGs), lack of per-beat annotated data and high waveform variability due to, among others, pathological conditions, uncertainty in lead positioning, body composition, gender or noisy recordings. This work attempts at helping boost research in the signal-based cardiovascular field by providing measurements over clinical data, facilitating further downstream tasks by augmenting clinical decision-making without providing black-box diagnostic solutions. By bridging the gap between the imaging and the signal communities for cardiovascular diseases, we demonstrate that a DL model, properly trained and with an adequate objective function, can provide good delineation with good generalization. The existing limitations hinder, however, the application of this model into the clinical practice. Besides the need for accessing a larger pool of data, prediction efficiency and model compression constraints must be met for its embedding in clinical systems. Possible directions for expansion would be to apply more extensive semi-supervised pre-training or data augmentation methodologies.

## Supplementary information

Supplementary Information.

## Data Availability

The dataset is publicly available at https://physionet.org/content/qtdb/1.0.0/.
