# Supplementary material for: Delineation of the electrocardiogram with a mixed-quality-annotations dataset using convolutional neural networks
Source: Sci Rep. 2021 Jan 13;11:863. doi: 10.1038/s41598-020-79512-7 (PMC7806759; doi:10.1038/s41598-020-79512-7)
Supplement: Supplementary file 1 — Supplementary Information. [file 41598_2020_79512_MOESM1_ESM.pdf]

Delineation of the Electrocardiogram with a Mixed-Quality-Annotations Dataset Using Convolutional Neural Networks

Supplementary Information

Guillermo Jimenez-Perez[1,*], Alejandro Alcaine[2,3,4], and Oscar Camara[1]

[1]PhySense research group, BCN-MedTech, Department of Information and Communication Technologies, Barcelona, 08018, Spain
[2]Facultad de Ciencias de la Salud, Universidad San Jorge, Zaragoza, 05830, Spain
[3]Biomedical Research Networking Center in Bioengineering, Biomaterials and Nanomedicine (CIBER-BBN), Madrid, 28029, Spain
[4]Biomedical Signal Interpretation and Computational Simulation (BSICoS) group, Arag´on Institute of Engineering Research, Zaragoza, 50018, Spain

[*]guillermo@jimenezperez.com

Spatial Dropout

Single-lead predictions

	ΔF1 (%)	Onset error (M+SD)	Offset error (M+SD)
P wave	6.03	-3.61 -6.61 ms	-5.43 -6.00 ms
QRS wave	11.23	-0.79 -3.28 ms	-0.22 -6.57 ms
T wave	14.35	**12.24 -0.39 ms**	-0.59 -11.59 ms

Table S1. Model performance comparison for a single-lead model with and without spatial dropout for the P, QRS and T waves. Positive F1 scores indicate performance gains, whereas negative onset or offset errors indicate less error. Values that perform worse have been bolded.

Multi-lead predictions

	ΔF1 (%)	Onset error (M+SD)	Offset error (M+SD)
P wave	1.38	-0.61+0.62 ms	-0.23+0.81 ms
QRS wave	3.62	1.40 -31.63 ms	3.39 -32.57 ms
T wave	9.85	**8.05+8.29 ms**	-1.31+3.52 ms

Table S2. Model performance comparison for a multi-lead model with and without spatial dropout for the P, QRS and T waves. Positive F1 scores indicate performance gains, whereas negative onset or offset errors indicate less error. Values that perform worse have been bolded.

Pre-training with low-quality labelling

	ΔF1 (%)	Onset error (M+SD)	Offset error (M+SD)
P wave	2.22	-3.14 -2.52 ms	-3.50 -2.29 ms
QRS wave	3.83	-0.59 -0.85 ms	-2.21 -2.86 ms
T wave	9.44	**7.93+3.57 ms**	-8.41 -6.11 ms

Table S3. Model performance comparison for a single-lead model pre-trained with low-quality labelling with and without spatial dropout for the P, QRS and T waves. Positive F1 scores indicate performance gains, whereas negative onset or offset errors indicate less error. Values that perform worse have been bolded.

Multi-lead pre-training with low-quality labelling

	ΔF1 (%)	Onset error (M+SD)	Offset error (M+SD)
P wave	0.28	**-2.30+6.91 ms**	**0.12+4.09 ms**
QRS wave	0.19	**-1.35+5.47 ms**	**4.48+7.42 ms**
T wave	0.83	**0.67+13.27 ms**	**-2.38+10.57 ms**

Table S4. Model performance comparison for a multi-lead model pre-trained with low-quality labelling with and without spatial dropout for the P, QRS and T waves. Positive F1 scores indicate performance gains, whereas negative onset or offset errors indicate less error. Values that perform worse have been bolded.

Pre-training with low-quality labelling

Single-lead without spatial dropout

	ΔF1 (%)	Onset error (M+SD)	Offset error (M+SD)
P wave	3.05	-0.29 - 6.16 ms	-0.39 -3.86 ms
QRS wave	7.90	-0.70 - 2.73 ms	0.31 -4.94 ms
T wave	0.97	2.71 - 4.31 ms	6.08 -12.98 ms

Table S5. Model performance comparison for a single-lead model without spatial dropout with and without pre-training with low-quality labellin g for the P, QRS and T waves. Positive F1 scores indicate performance g ains, whereas negative onset or offset errors indicate less error. Valu es that perform worse have been bolded.

Multi-lead without spatial dropout

	ΔF1 (%)	Onset error (M+SD)	Offset error (M+SD)
P wave	4.75	-0.46 -11.03 ms	-1.47 -6.49 ms
QRS wave	5.51	2.79 -37.51 ms	-0.31 -35.55 ms
T wave	10.40	9.22 -7.49 ms	-3.39 -7.35 ms

Table S6. Model performance comparison for a multi-lead model without s patial dropout with and without pre-training with low-quality labelling for the P, QRS and T waves. Positive F1 scores indicate performance gai ns, whereas negative onset or offset errors indicate less error. Values that perform worse have been bolded.

Single-lead with spatial dropout

	ΔF1 (%)	Onset error (M+SD)	Offset error (M+SD)
P wave	1.38	-0.20 -3.46 ms	-0.13 -2.12 ms
QRS wave	0.29	-0.62 -0.67 ms	-0.30 -0.60 ms
T wave	0.77	0.96 -2.93 ms	-0.96 -3.13 ms

Table S7. Model performance comparison for a single-lead model with spa tial dropout with and without pre-training with low-quality labelling f or the P, QRS and T waves. Positive F1 scores indicate performance gain s, whereas negative onset or offset errors indicate less error. Values that perform worse have been bolded.

Multi-lead with spatial dropout

	ΔF1 (%)	Onset error (M+SD)	Offset error (M+SD)
P wave	2.52	-0.71 -4.61 ms	-1.63 -2.08 ms
QRS wave	1.70	**0.29+2.25 ms**	1.14 -0.80 ms
T wave	2.61	4.88 -4.62 ms	-1.95 -9.33 ms

Table S8. Model performance comparison for a multi-lead model with spat ial dropout with and without pre-training with low-quality labelling fo r the P, QRS and T waves. Positive F1 scores indicate performance gains , whereas negative onset or offset errors indicate less error. Values t hat perform worse have been bolded.

Single lead vs. Multi-lead

High-quality annotations (without spatial dropout)

	ΔF1 (%)	Onset error (M+SD)	Offset error (M+SD)
P wave	3.47	**-1.04+0.81 ms**	-3.95 -0.32 ms
QRS wave	7.67	**-0.44+27.04 ms**	**-1.34+19.73 ms**
T wave	4.92	**-5.30+6.96 ms**	0.15 -2.88 ms

Table S9. Model performance comparison of high-quality annotations with out spatial dropout with single- and multi-lead strategies for the P, Q RS and T waves. Positive F1 scores indicate performance gains, whereas negative onset or offset errors indicate less error. Values that perfor m worse have been bolded.

Pre-training with low-quality annotations (without spatial dropout)

	ΔF1 (%)	Onset error (M+SD)	Offset error (M+SD)
P wave	1.01	1.09 -2.41 ms	**-3.56+0.55 ms**
QRS wave	3.12	**2.70+0.41 ms**	-3.69 -0.01 ms
T wave	8.81	**1.36+6.28 ms**	**-9.19+1.82 ms**

Table S10. Model performance comparison of a model pre-trained with low -quality annotations without spatial dropout with single- and multi-lea d strategies for the P, QRS and T waves. Positive F1 scores indicate pe rformance gains, whereas negative onset or offset errors indicate less error. Values that perform worse have been bolded.

High-quality annotations (with spatial dropout)

	ΔF1 (%)	Onset error (M+SD)	Offset error (M+SD)
P wave	**-2.39**	**0.90+10.27 ms**	**1.57+5.81 ms**
QRS wave	**-0.70**	**1.34+4.84 ms**	**2.74+8.73 ms**
T wave	**-1.60**	**-7.61+16.03 ms**	**0.22+19.00 ms**

Table S11. Model performance comparison of high-quality annotations wit h spatial dropout with single- and multi-lead strategies for the P, QRS and T waves. Positive F1 scores indicate performance gains, whereas neg ative onset or offset errors indicate less error. Values that perform w orse have been bolded.

Pre-training with low-quality annotations (with spatial dropout)

	ΔF1 (%)	Onset error (M+SD)	Offset error (M+SD)
P wave	**-0.67**	**0.95+7.84 ms**	**0.09+6.26 ms**
QRS wave	0.19	**2.31+7.37 ms**	**2.88+9.05 ms**
T wave	0.02	**-4.52+14.66 ms**	**-1.67+15.75 ms**

Table S12. Model performance comparison of a model pre-trained with low -quality annotations with spatial dropout with single- and multi-lead s trategies for the P, QRS and T waves. Positive F1 scores indicate perfo rmance gains, whereas negative onset or offset errors indicate less err or. Values that perform worse have been bolded.

Model depth

1. F1 SCORE

P wave – All executions

Depth	4	5	6	7
4	0	**-0.01**	**-1.85**	**-5.92**
5		0	**-1.50**	**-3.42**
6			0	**-3.48**
7				0

Table S13. Model F1 score comparison of models with different depths for models with and without spatial dropout for the P wave. Positive F1 scores indicate performance gains. Values that perform worse have been bolded.

QRS wave – All executions

Depth	4	5	6	7
4	0	**-0.05**	**-0.07**	**-1.49**
5		0	0.05	**-0.97**
6			0	**-0.14**
7				0

Table S14. Model F1 score comparison of models with different depths for models with and without spatial dropout for the QRS wave. Positive F1 scores indicate performance gains. Values that perform worse have been bolded.

T wave – All executions

Depth	4	5	6	7
4	0	**-0.19**	0.93	**-4.15**
5		0	**-0.84**	**-2.55**
6			0	**-1.61**
7				0

Table S15. Model F1 score comparison of models with different depths for models with and without spatial dropout for the T wave. Positive F1 scores indicate performance gains. Values that perform worse have been bolded.

2. ONSET ERROR

P wave – All executions

Depth	4	5	6	7
4	0+0	**-1.04+4.05**	**-0.21+3.62**	**-1.84+3.56**
5		**0+0**	0.27 -0.35	**-1.77+1.45**
6			**0+0**	-0.90-2.06
7				0+0

Table S16. Model onset error comparison of models with different depths for models with and without spatial dropout for the P wave. Negative on set errors indicate performance gains. Values that perform worse have b een bolded.

QRS wave – All executions

Depth	4	5	6	7
4	0+0	-0.31 -0.22	**-0.30+0.40**	-0.70+0.07
5		0+0	**-0.01+0.68**	-0.67-0.16
6			0+0	-0.50-0.27
7				0+0

Table S17. Model onset error comparison of models with different depths for models with and without spatial dropout for the QRS wave. Negative onset errors indicate performance gains. Values that perform worse have been bolded.

T wave – All executions

Depth	4	5	6	7
4	0+0	**-0.11+4.07**	**-3.58+6.50**	**1.52+4.37**
5		0+0	**-4.37+2.92**	**-1.04+2.15**
6			0+0	4.47-4.51
7				0+0

Table S18. Model onset error comparison of models with different depths for models with and without spatial dropout for the T wave. Negative on set errors indicate performance gains. Values that perform worse have b een bolded.

3. OFFSET ERROR

P wave – All executions

Depth	4	5	6	7
4	0+0	**-1.46+2.86**	**0.51+3.53**	**-0.34+2.66**
5		0+0	**1.74+0.03**	-1.22-0.01
6			0+0	-0.98-2.59
7				0+0

Table S19. Model offset error comparison of models with different depths for models with and without spatial dropout for the P wave. Negative offset errors indicate performance gains. Values that perform worse have been bolded.

QRS wave – All executions

Depth	4	5	6	7
4	0+0	0.03 -0.32	**-0.93+1.56**	**0.70-0.10**
5		0+0	**-0.59+0.24**	**0.67+0.73**
6			0+0	0.47-1.87
7				0+0

Table S20. Model onset error comparison of models with different depths for models with and without spatial dropout for the QRS wave. Negative offset errors indicate performance gains. Values that perform worse have been bolded.

T wave – All executions

Depth	4	5	6	7
4	0+0	**1.86+5.80**	**1.54+7.81**	**0.19+5.96**
5		0+0	**1.85+5.41**	**-3.88+2.19**
6			0+0	**-4.97+1.38**
7				0+0

Table S21. Model onset error comparison of models with different depths for models with and without spatial dropout for the T wave. Negative offset errors indicate performance gains. Values that perform worse have been bolded.

Model repetitions

1. F1 SCORE

P wave – All executions

Repetitions	2	3	4
2	0+0	0.32	**-10.70**
3		0+0	**-7.15**
4			0+0

Table S22. Model F1 score comparison of models with different number of module repetitions for models with and without spatial dropout for the P wave. Positive F1 scores indicate performance gains. Values that perf orm worse have been bolded.

QRS wave – All executions

Repetitions	2	3	4
2	0+0	**-0.07**	**-4.09**
3		0+0	**-2.96**
4			0+0

Table S23. Model F1 score comparison of models with different number of module repetitions for models with and without spatial dropout for the QRS wave. Positive F1 scores indicate performance gains. Values that pe rform worse have been bolded.

T wave – All executions

Repetitions	2	3	4
2	0+0	1.63	**-3.10**
3		0+0	**-5.36**
4			0+0

Table S24. Model F1 score comparison of models with different number of module repetitions for models with and without spatial dropout for the T wave. Positive F1 scores indicate performance gains. Values that perf orm worse have been bolded.

2. ONSET ERROR

P wave – All executions

Repetitions	2	3	4
2	0+0	**0.47+0.97**	**-0.44+3.73**
3		0+0	**0.42+1.33**
4			0+0

Table S25. Model onset error comparison of models with different number of module repetitions for models with and without spatial dropout for t he P wave. Negative onset errors indicate performance gains. Values tha t perform worse have been bolded.

QRS wave - All executions

Repetitions	2	3	4
2	0+0	-0.48-0.36	-2.42-1.25
3		0+0	-1.39+0.38
4			0+0

Table S26. Model onset error comparison of models with different number of module repetitions for models with and without spatial dropout for the QRS wave. Negative onset errors indicate performance gains. Values that perform worse have been bolded.

T wave - All executions

Repetitions	2	3	4
2	0+0	**7.31+3.35**	**7.31+3.35**
3		0+0	**14.46+2.52**
4			0+0

Table S27. Model onset error comparison of models with different number of module repetitions for models with and without spatial dropout for the T wave. Negative onset errors indicate performance gains. Values that perform worse have been bolded.

3. OFFSET ERROR

P wave - All executions

Repetitions	2	3	4
2	0+0	**-0.53+1.34**	**0.61+1.17**
3		0+0	1.82-2.15
4			0+0

Table S28. Model offset error comparison of models with different number of module repetitions for models with and without spatial dropout for the P wave. Negative offset errors indicate performance gains. Values that perform worse have been bolded.

QRS wave - All executions

Repetitions	2	3	4
2	0+0	**0.94+1.62**	**3.18+1.11**
3		0+0	**0.77+0.13**
4			0+0

Table S29. Model offset error comparison of models with different number of module repetitions for models with and without spatial dropout for the QRS wave. Negative offset errors indicate performance gains. Values that perform worse have been bolded.

T wave – All executions

Repetitions	2	3	4
2	0+0	-1.75-0.96	**-4.98+18.33**
3		0+0	**-3.70+18.69**
4			0+0

Table S30. Model offset error comparison of models with different number of module repetitions for models with and without spatial dropout for the T wave. Negative offset errors indicate performance gains. Values that perform worse have been bolded.

Convolutional Module

1. F1 SCORE

P wave – All executions

Module	Vanilla	Residual	XCeption
Vanilla	0	**-1.20**	0.11
Residual		0	0.74
XCeption			0

Table S31. Model F1 score comparison of models with different convolutional operations for the P wave. Positive F1 scores indicate performance gains. Values that perform worse have been bolded.

QRS wave – All executions

Module	Vanilla	Residual	XCeption
Vanilla	0	**-0.25**	0.08
Residual		0	0.22
XCeption			0

Table S32. Model F1 score comparison of models with different convolutional operations for the QRS wave. Positive F1 scores indicate performance gains. Values that perform worse have been bolded.

T wave – All executions

Module	Vanilla	Residual	XCeption
Vanilla	0	**-0.87**	**-0.45**
Residual		0	0.64
XCeption			0

Table S33. Model F1 score comparison of models with different convolutional operations for the T wave. Positive F1 scores indicate performance gains. Values that perform worse have been bolded.

2. ONSET ERROR

P wave – All executions

Module	Vanilla	Residual	XCeption
Vanilla	0+0	-1.33-4.44	-0.72-4.16
Residual		0+0	0.59-0.10
XCeption			0+0

Table S34. Model onset error comparison of models with different convol utional operations for the P wave. Negative onset errors indicate perfo rmance gains. Values that perform worse have been bolded.

QRS wave – All executions

Module	Vanilla	Residual	XCeption
Vanilla	0+0	0.80-0.35	0.42-1.30
Residual		0+0	-0.60+0.02
XCeption			0+0

Table S35. Model onset error comparison of models with different convol utional operations for the P wave. Negative onset errors indicate perfo rmance gains. Values that perform worse have been bolded.

T wave – All executions

Module	Vanilla	Residual	XCeption
Vanilla	0+0	-6.90-3.43	-11.45-3.08
Residual		0+0	-2.54-4.47
XCeption			0+0

Table S36. Model onset error comparison of models with different convol utional operations for the P wave. Negative onset errors indicate perfo rmance gains. Values that perform worse have been bolded.

3. OFFSET ERROR

P wave – All executions

Module	Vanilla	Residual	XCeption
Vanilla	0+0	2.15-2.19	2.96-2.18
Residual		0+0	**1.47+0.13**
XCeption			0+0

Table S37. Model offset error comparison of models with different convo lutional operations for the P wave. Negative offset errors indicate per formance gains. Values that perform worse have been bolded.

QRS wave – All executions

Module	Vanilla	Residual	XCeption
Vanilla	0+0	-0.29-1.07	-1.15-1.71
Residual		0+0	-0.44-1.52
XCeption			0+0

Table S38. Model offset error comparison of models with different convo lutional operations for the QRS wave. Negative offset errors indicate p erformance gains. Values that perform worse have been bolded.

T wave – All executions

Module	Vanilla	Residual	XCeption
Vanilla	0+0	-1.41-1.78	2.95-1.75
Residual		0+0	2.67-0.65
XCeption			0+0

Table S39. Model offset error comparison of models with different convo lutional operations for the T wave. Negative offset errors indicate per formance gains. Values that perform worse have been bolded.

Predictions on an external dataset

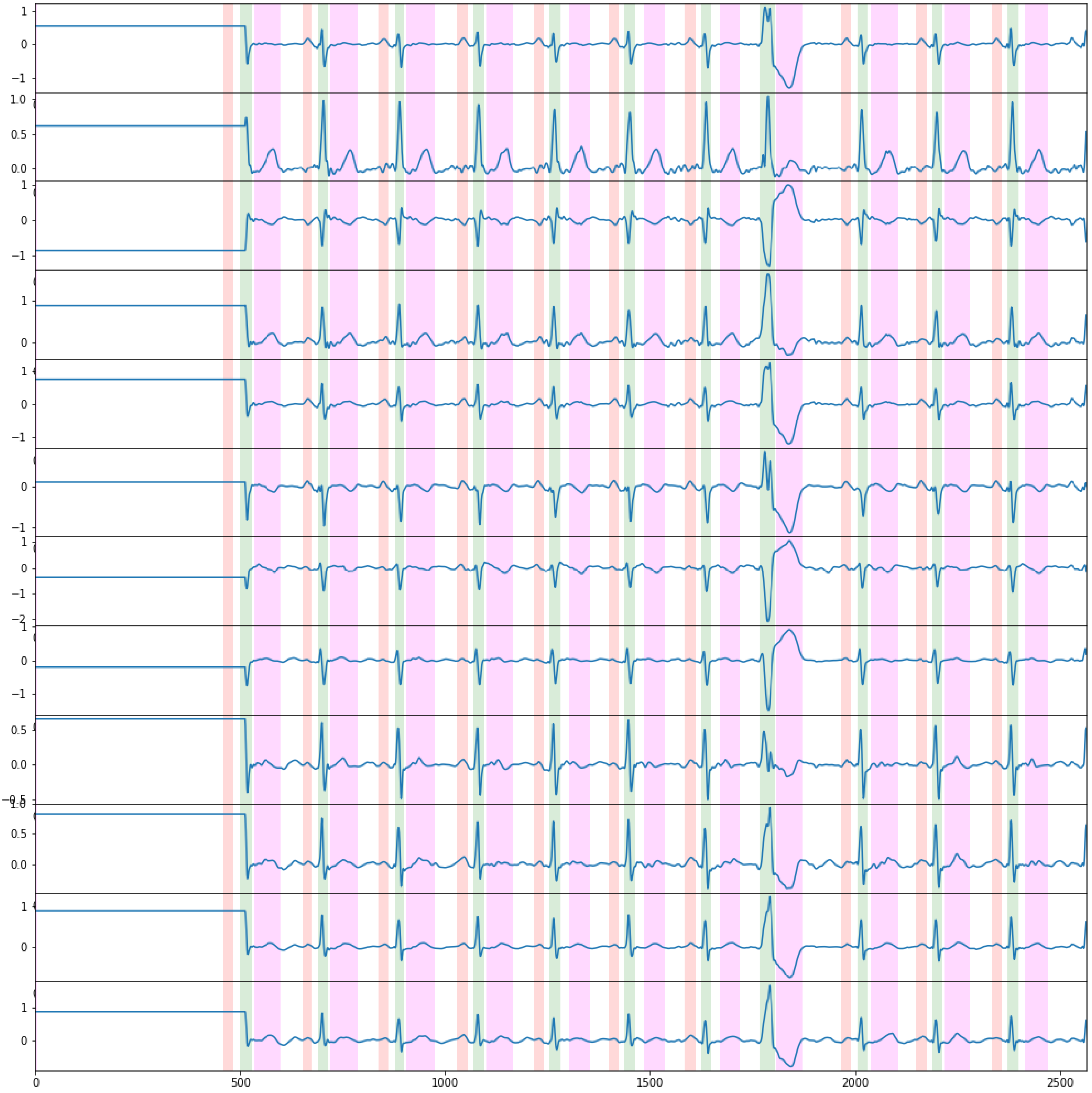

Figure S1: Algorithm's prediction in the recording 1010240 from Zheng, J., Fu, G., Anderson, K. *et al*. A 12-Lead ECG database to identify origins of idiopathic ventricular arrhythmia containing 334 patients. *Sci Data* **7,** 98 (2020). https://doi.org/10.1038/s41597-020-0440-8. The beat fusion has been produced by agreement between, at least, 25% of the leads.

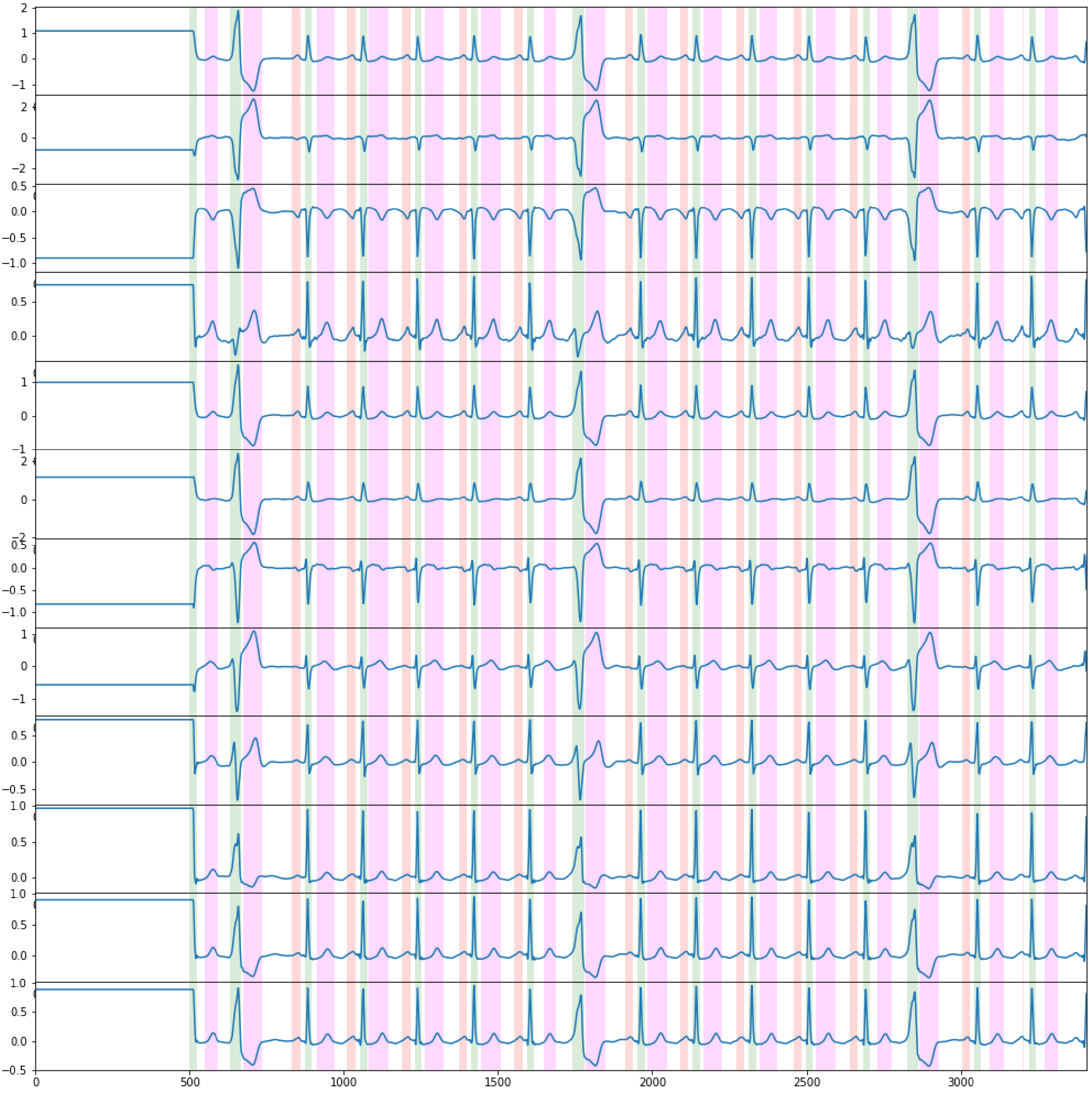

Figure S2: Algorithm's prediction in the recording 1014570 from Zheng, J., Fu, G., Anderson, K. *et al*. A 12-Lead ECG database to identify origins of idiopathic ventricular arrhythmia containing 334 patients. *Sci Data* **7,** 98 (2020). https://doi.org/10.1038/s41597-020-0440-8. The beat fusion has been produced by agreement between, at least, 25% of the leads.

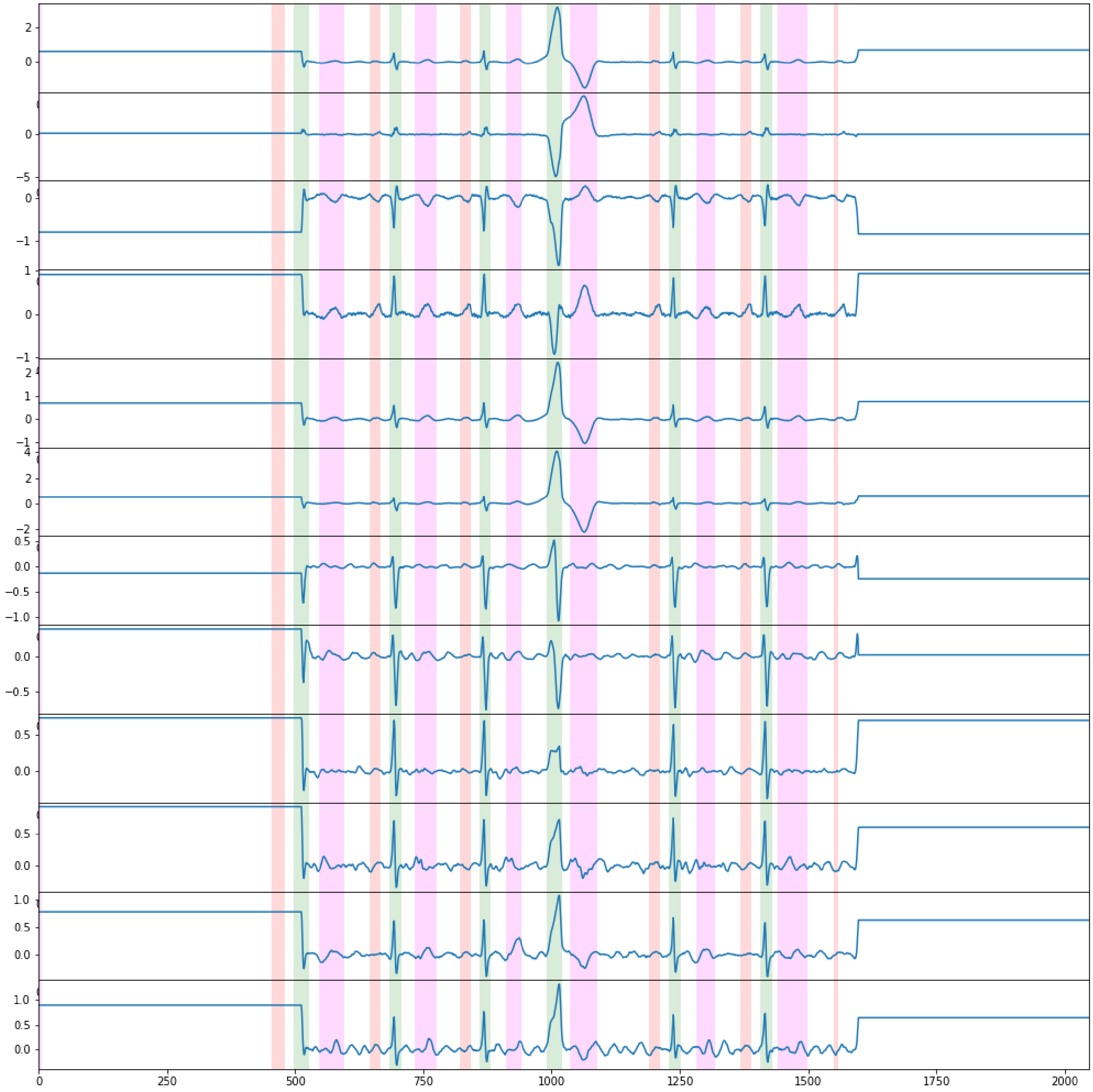

Figure S3: Algorithm's prediction in the recording 1000364 from Zheng, J., Fu, G., Anderson, K. *et al*. A 12-Lead ECG database to identify origins of idiopathic ventricular arrhythmia containing 334 patients. *Sci Data* **7,** 98 (2020). https://doi.org/10.1038/s41597-020-0440-8. The beat fusion has been produced by agreement between, at least, 25% of the leads.

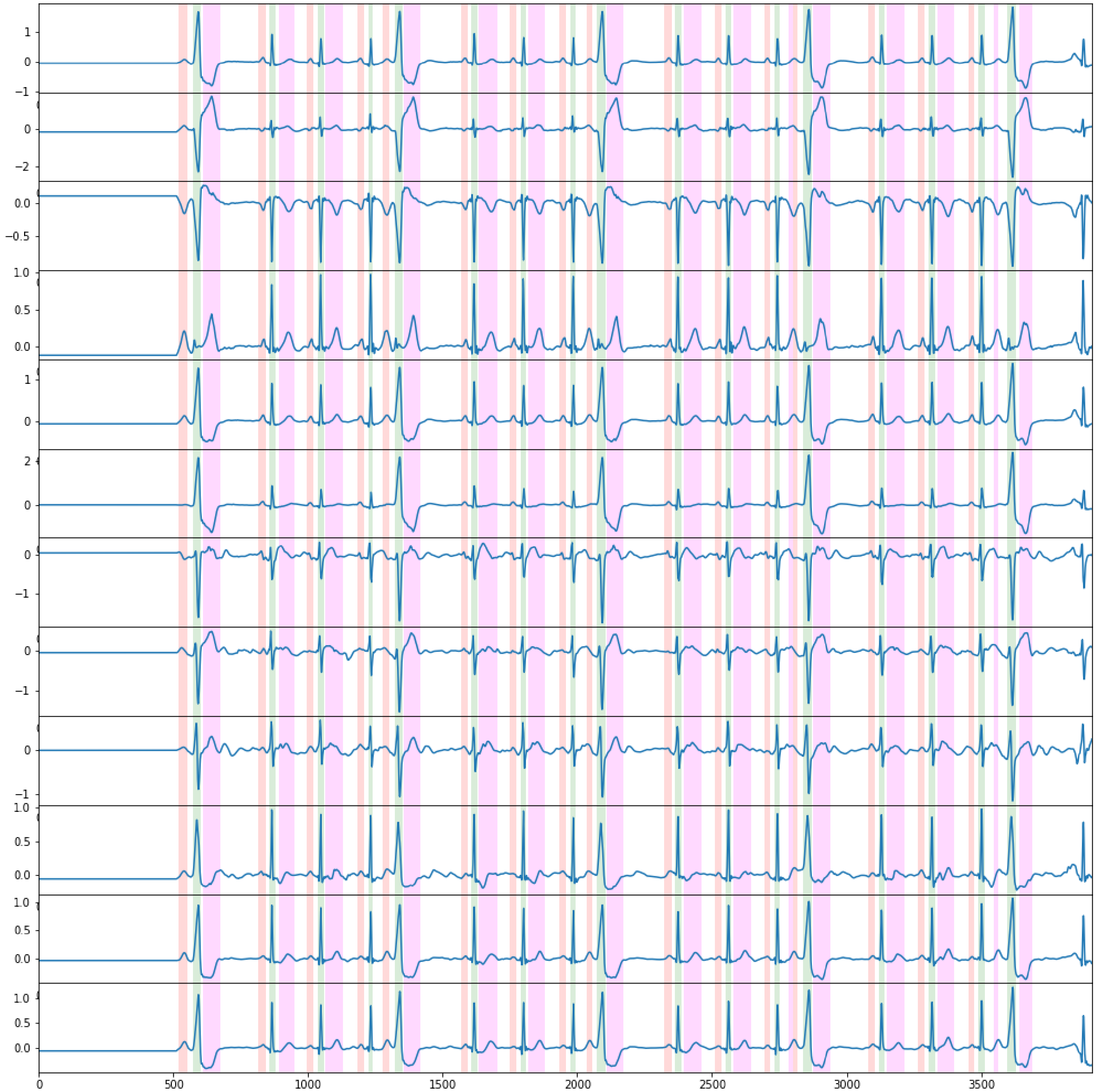

Figure S4: Algorithm's prediction in the recording 1015792 from Zheng, J., Fu, G., Anderson, K. *et al*. A 12-Lead ECG database to identify origins of idiopathic ventricular arrhythmia containing 334 patients. *Sci Data* **7,** 98 (2020). https://doi.org/10.1038/s41597-020-0440-8. The beat fusion has been produced by agreement between, at least, 25% of the leads.

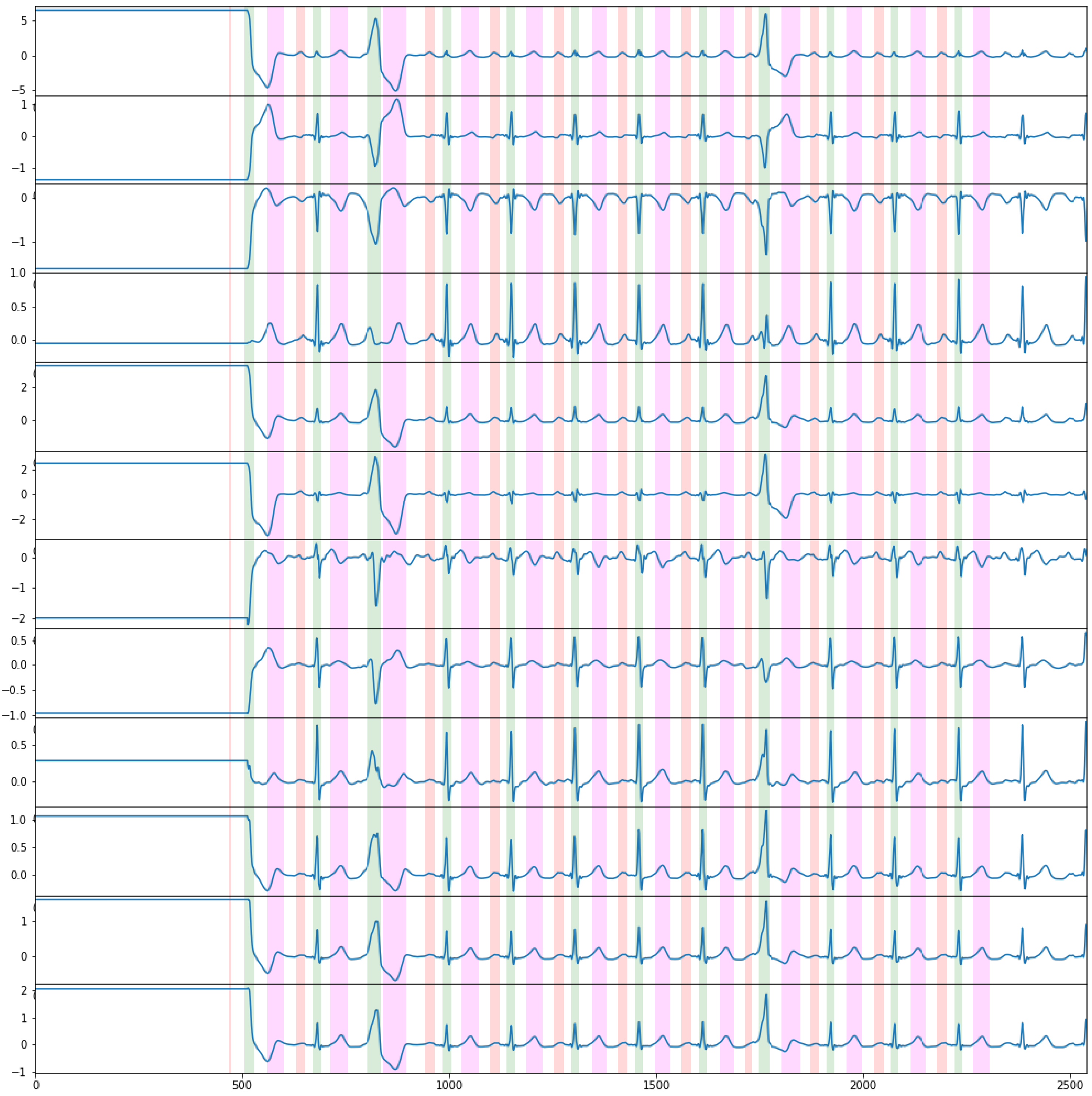

Figure S5: Algorithm's prediction in the recording 1016888 from Zheng, J., Fu, G., Anderson, K. *et al*. A 12-Lead ECG database to identify origins of idiopathic ventricular arrhythmia containing 334 patients. *Sci Data* **7,** 98 (2020). https://doi.org/10.1038/s41597-020-0440-8. The beat fusion has been produced by agreement between, at least, 25% of the leads.

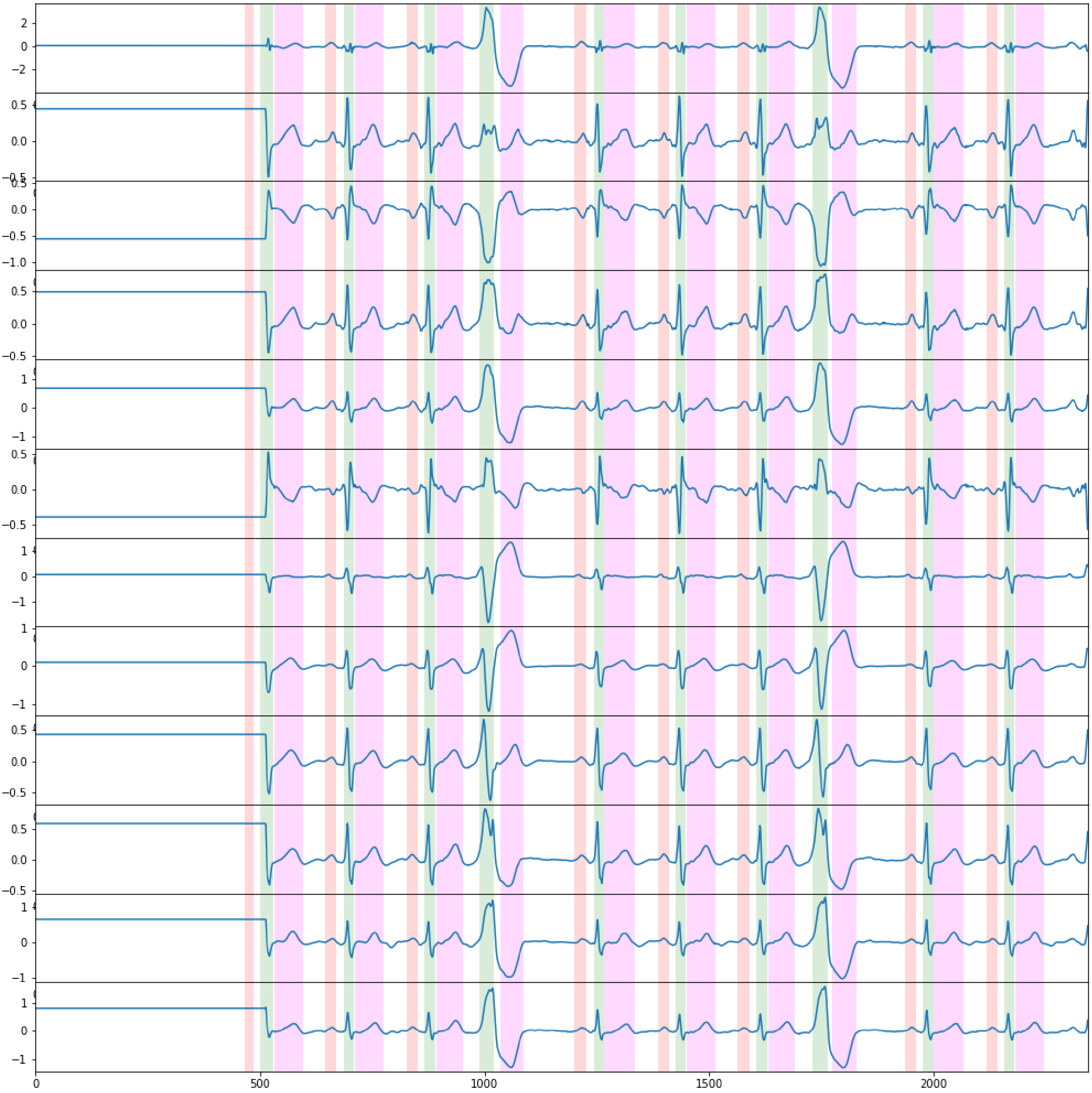

Figure S6: Algorithm's prediction in the recording 707139 from Zheng, J., Fu, G., Anderson, K. *et al*. A 12-Lead ECG database to identify origins of idiopathic ventricular arrhythmia containing 334 patients. *Sci Data* **7,** 98 (2020). https://doi.org/10.1038/s41597-020-0440-8. The beat fusion has been produced by agreement between, at least, 25% of the leads.

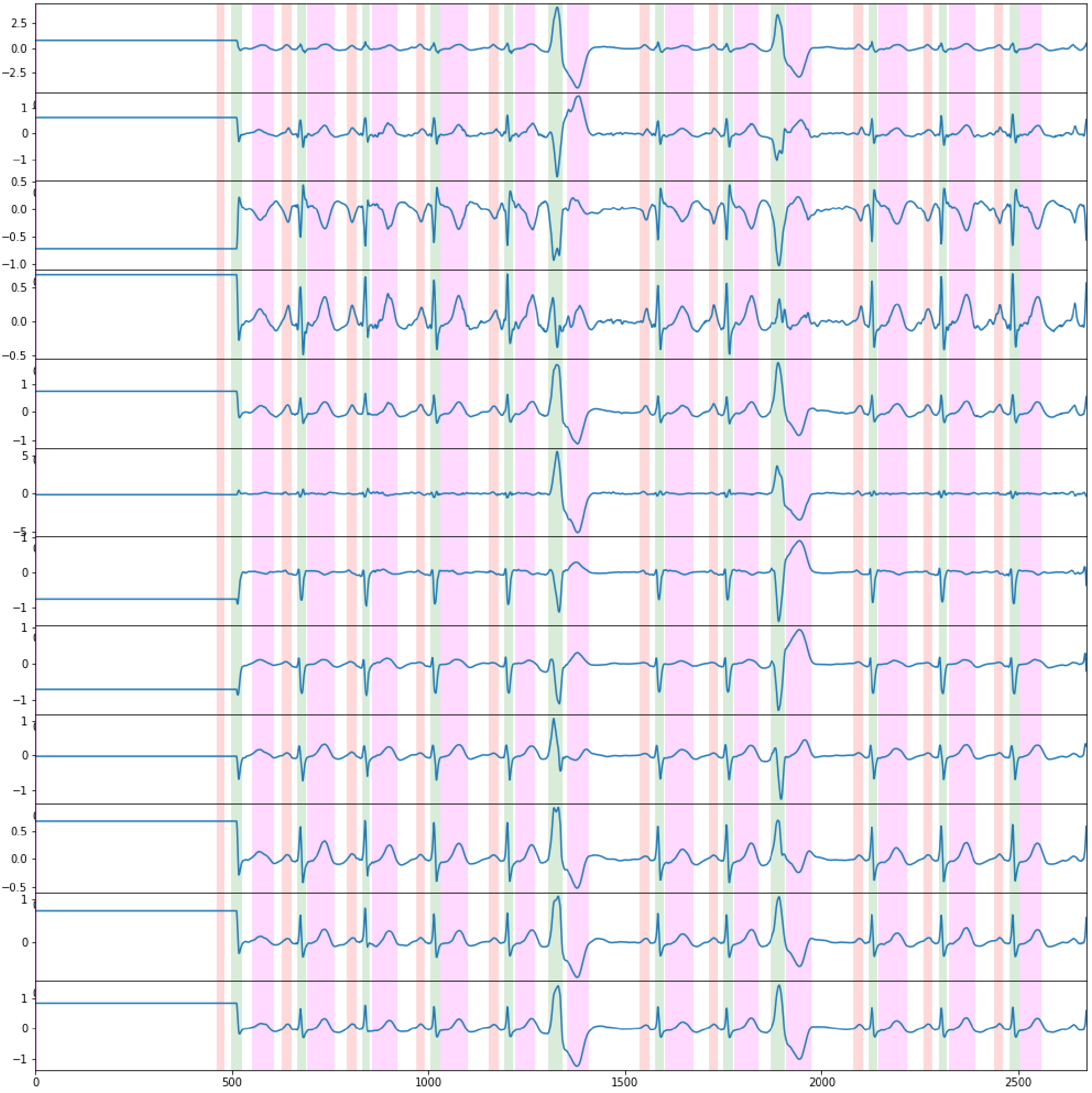

Figure S7: Algorithm's prediction in the recording 708534 from Zheng, J., Fu, G., Anderson, K. *et al*. A 12-Lead ECG database to identify origins of idiopathic ventricular arrhythmia containing 334 patients. *Sci Data* **7,** 98 (2020). https://doi.org/10.1038/s41597-020-0440-8. The beat fusion has been produced by agreement between, at least, 25% of the leads.

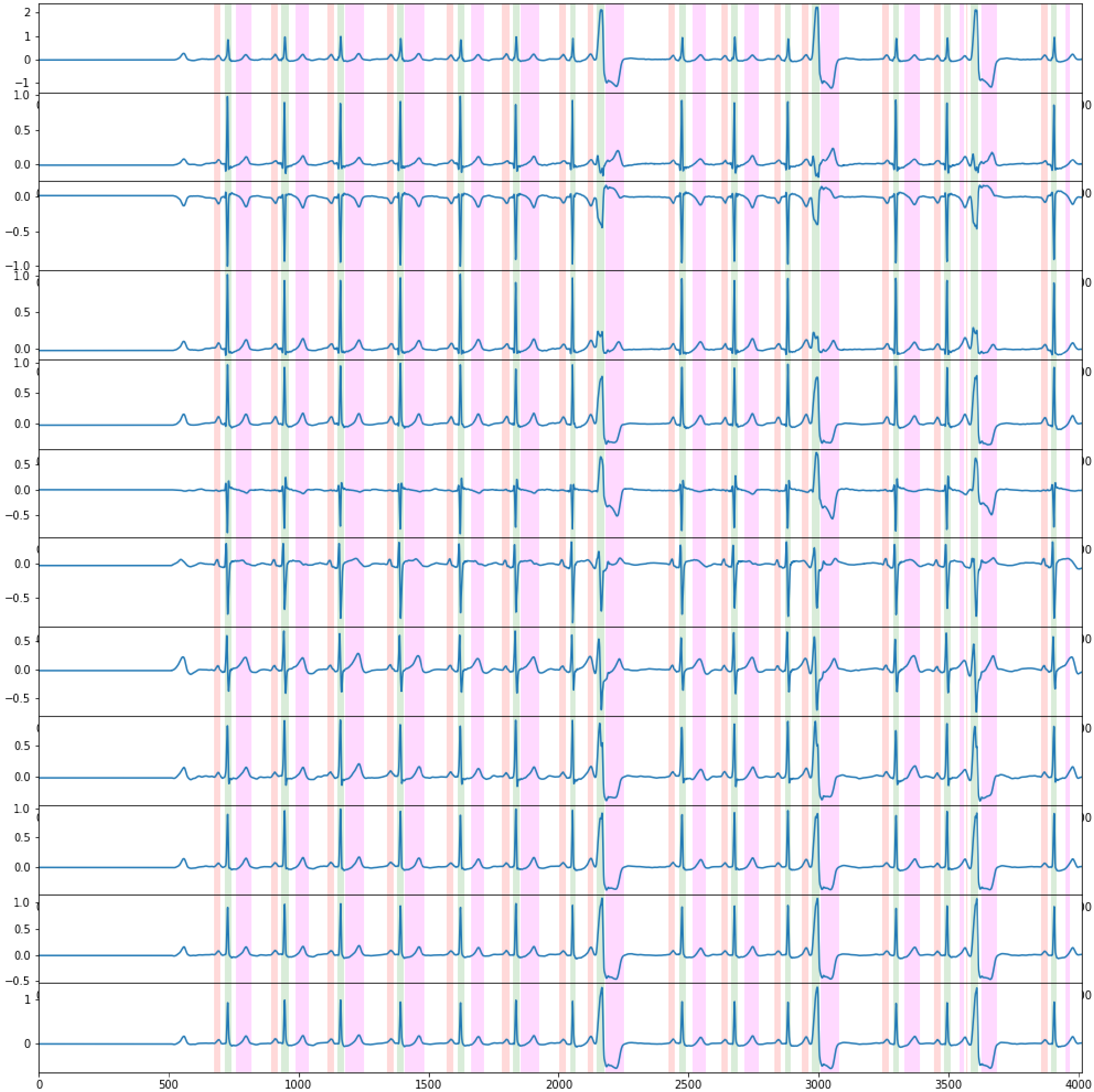

Figure S8: Algorithm's prediction in the recording 757884 from Zheng, J., Fu, G., Anderson, K. *et al*. A 12-Lead ECG database to identify origins of idiopathic ventricular arrhythmia containing 334 patients. *Sci Data* **7,** 98 (2020). https://doi.org/10.1038/s41597-020-0440-8. The beat fusion has been produced by agreement between, at least, 25% of the leads.

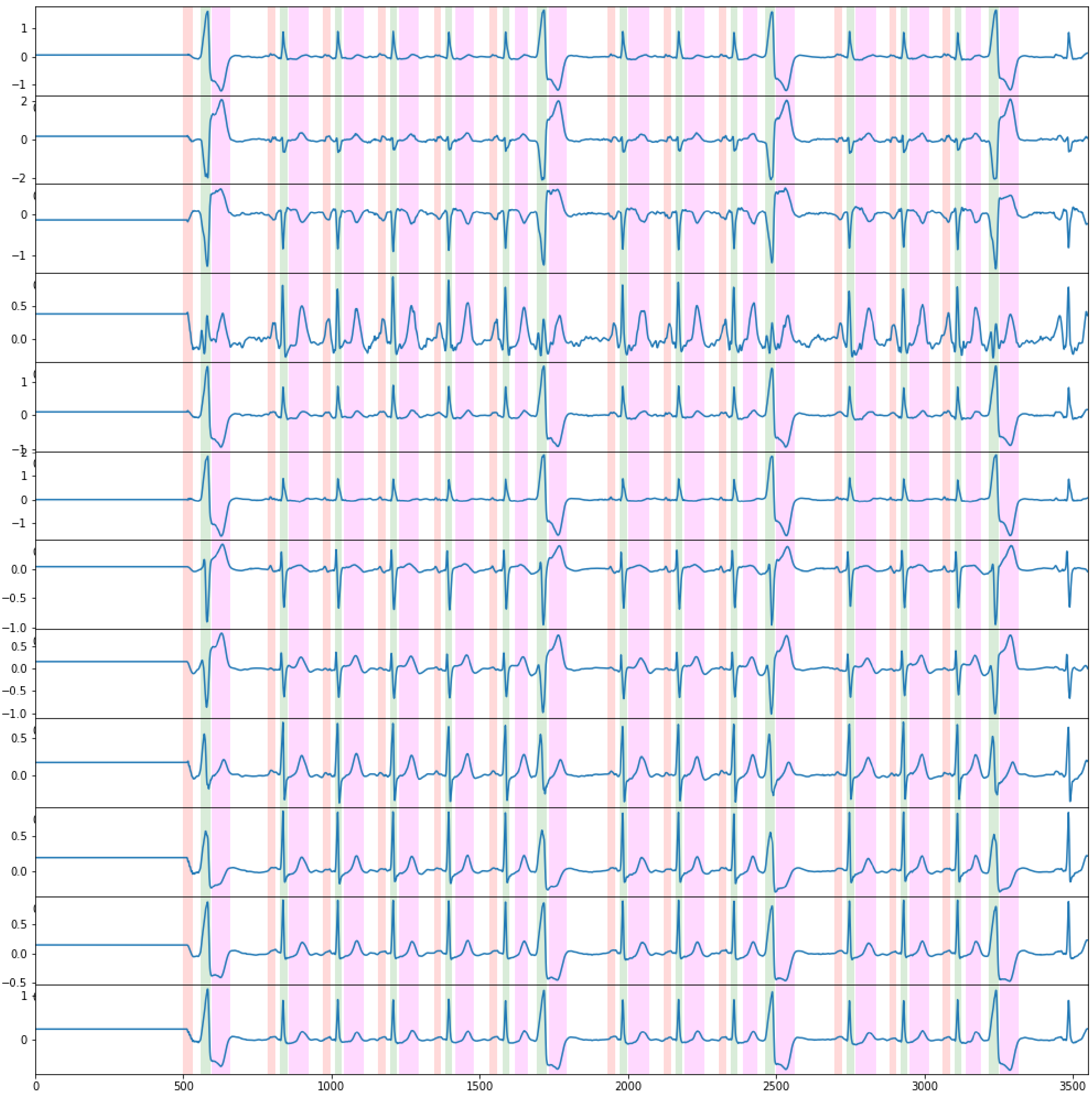

Figure S9: Algorithm's prediction in the recording 1033211 from Zheng, J., Fu, G., Anderson, K. *et al*. A 12-Lead ECG database to identify origins of idiopathic ventricular arrhythmia containing 334 patients. *Sci Data* **7,** 98 (2020). https://doi.org/10.1038/s41597-020-0440-8. The beat fusion has been produced by agreement between, at least, 25% of the leads.

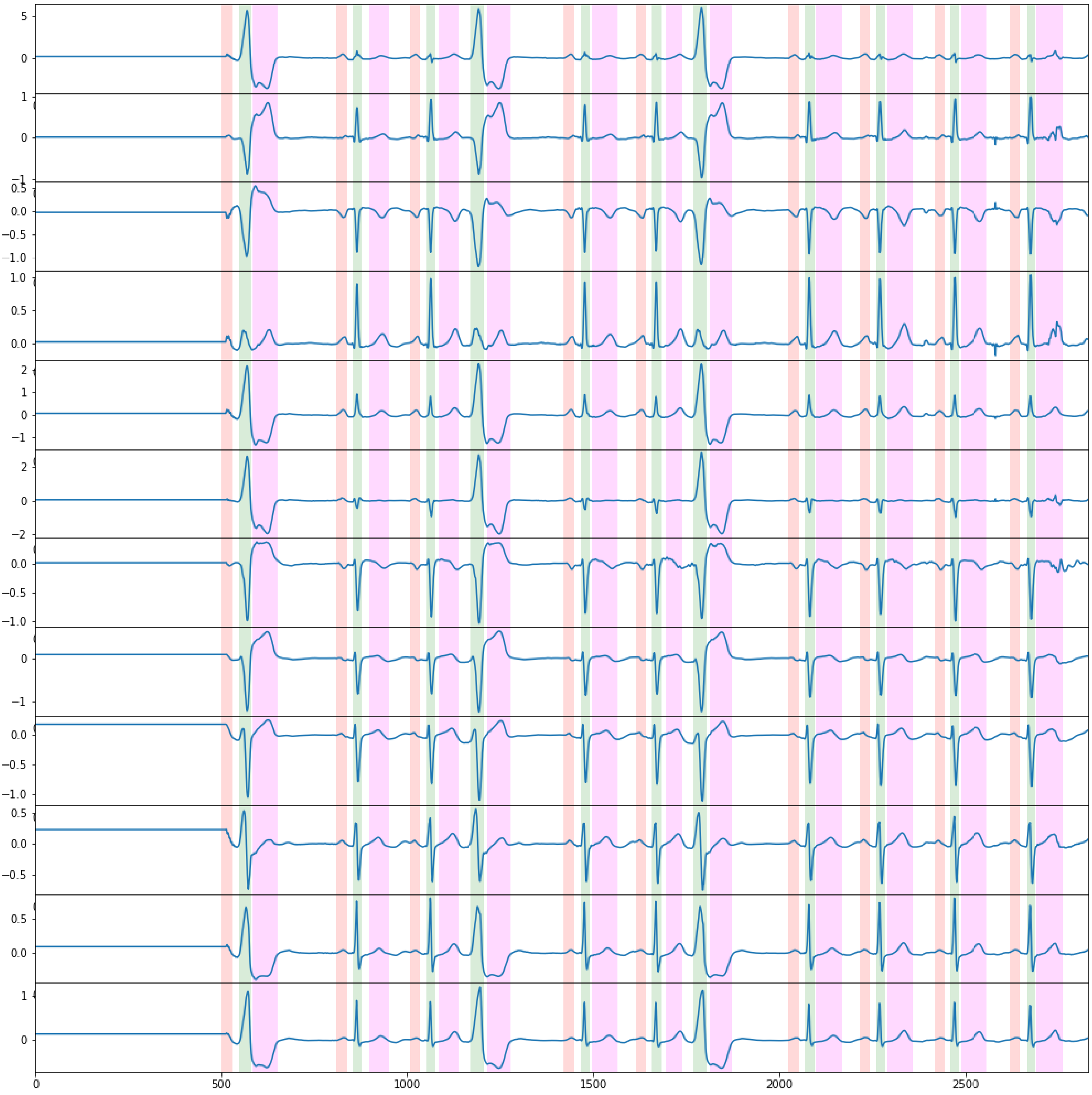

Figure S10: Algorithm's prediction in the recording 1067472 from Zheng, J., Fu, G., Anderson, K. *et al*. A 12-Lead ECG database to identify origins of idiopathic ventricular arrhythmia containing 334 patients. *Sci Data* **7,** 98 (2020). https://doi.org/10.1038/s41597-020-0440-8. The beat fusion has been produced by agreement between, at least, 25% of the leads.
